# Effect of Caffeine and Nitrates Combination on Exercise Performance, Heart Rate and Oxygen Uptake: A Systematic Review and Meta-Analysis

**DOI:** 10.3390/nu16193352

**Published:** 2024-10-02

**Authors:** Laura Gilsanz, Juan Del Coso, Sergio L. Jiménez-Saiz, Helios Pareja-Galeano

**Affiliations:** 1 Department of Physical Education, Sport and Human Movement, Universidad Autónoma de Madrid, 28049 Madrid, Spain; laura.gilsanz@estudiante.uam.es; 2 Sport Sciences Research Centre, Universidad Rey Juan Carlos, Fuenlabrada, 28942 Madrid, Spain; juan.delcoso@urjc.es (J.D.C.); sergio.jimenez.saiz@urjc.es (S.L.J.-S.)

**Keywords:** caffeine, nitrates, beetroot juice, co-supplementation, performance

## Abstract

Background: The evidence about the synergy of combining caffeine (CAF) and nitrates on exercise performance has not been summarized, although there is a possibility of additive/synergistic effects of the co-ingestion of these substances given their different mechanisms of action in central (CAF) and peripheral tissues (nitrates). Objectives: The aim was to analyze the effects of co-supplementation of CAF and nitrates on sports performance in comparison to the isolated ingestion of these substances. Methods: The databases of PubMed, Web of Science, Medline, CiNAHL and SPORTDiscus were used until June 2024 following PRISMA guidelines. Randomized controlled trials, at least one single-blind trial, conducted in adults were considered. A meta-analysis was performed using the random effects model to calculate the standardized mean difference estimated by Hedges’ *g* and 95% confidence intervals (CIs) for studies with four arms. Results: Six studies were included (N = 95). The meta-analysis revealed that caffeine and nitrates supplementation (CAF+nitrates) did not enhance performance in time trials (TTs) over the CAF alone (*g* = −0.06; 95% CI = −0.46 to 0.35; *p* = 0.78) or nitrates alone (*g* = 0.29; 95% CI = −0.12 to 0.70; *p* = 0.17). CAF+nitrates did not affect heart rate during submaximal exercise trials over CAF alone (*g* = 0.04; 95% CI = −0.31 to 0.40; *p* = 0.80) or nitrates alone (*g* = −0.15; 95% CI = −0.50 to 0.20; *p* = 0.40). Likewise, CAF+nitrates did not affect oxygen uptake during submaximal exercise trials over CAF alone (*g* = −0.04; 95% CI = −0.45 to 0.37; *p* = 0.84) or nitrates alone (*g* = −0.29; 95% CI = −0.70 to 0.12; *p* = 0.16). Conclusions: CAF+nitrates did not offer further benefits on exercise performance or physiological variables from the isolated intake of CAF and nitrates.

## 1. Introduction

The co-supplementation of ergogenic aids in sports has become a common strategy, and although a synergistic effect is expected, some combinations can have neutral or even ergolytic results [1].

An example of an additional performance-enhancing effect is co-ingestion of β-alanine and sodium bicarbonate (SB). These produce an additional reduction in fatigue caused by muscle acidosis through the combination of intracellular (β-alanine) and extracellular (SB) buffer systems [1,2]. Similarly, the combination of β-alanine with creatine has been shown to have an additive effect on performance improvement, by enhancing the carnosine and phosphocreatine buffer systems [1].

However, other combinations of supplements do not demonstrate synergistic effects, for example, the co-ingestion of caffeine (CAF) and creatine [3]. In certain instances, the combination of supplements can even result in an increased incidence and severity of side effects. This is exemplified by the combination of SB and caffeine, which has been observed to elevate the likelihood of gastrointestinal discomfort [4].

CAF and nitrates are classified as Group A by Australian Institute of Sport (AIS). This group includes supplements with “sound scientific evidence for use in specific situations in sport using evidence-based protocols” [5]. CAF operates as an antagonist of adenosine receptors, inhibiting the “fatiguing” effect of adenosine during exercise [6]. Inorganic nitrates are involved in the synthesis of nitric oxide (NO) and it has been observed that their consumption prior to endurance exercise can reduce oxygen consumption during exercise and improve time to exhaustion, cardiorespiratory performance at the anaerobic threshold and maximum oxygen uptake (VO_2max_).

Despite the popularity of these substances in sports contexts, the evidence about the synergies of combining CAF and nitrates on exercise performance has not been summarized and meta-analyzed. Given different mechanisms of action: central (CAF) versus peripheral (nitrates) and the fact that CAF has not demonstrated an effect on NO, at least in rodents [7], it is possible that co-ingestion of these substances together may have synergistic effects on performance. Therefore, this meta-analysis aims to evaluate the effects of co-supplementation of caffeine and nitrates on sports performance, comparing it to the isolated intake of these substances. This study seeks to determine whether the combination of caffeine and nitrates produces significant improvements in aerobic performance, such as VO_2max_ and heart rate.

## 2. Materials and Methods

For this systematic review, we followed Preferred Reporting Items for Systematic Reviews and Meta-analysis (PRISMA) guidelines [8].

### 2.1. Inclusion/Exclusion Criteria

Eligibility criteria for study selection were those of the PICOS model: population, intervention, comparison, outcomes and study design. The study population included healthy men and women over 18 years. The intervention considered was the administration of CAF and nitrates supplementations (CAF+nitrates) together before an exercise bout. As comparators, we established a comparison of the effect of CAF+nitrates with the intake of CAF alone, nitrates alone and a placebo (PLA). We considered all articles that fulfilled these criteria irrespective of the dose of caffeine and nitrates administered when the dose of these substances in the CAF+nitrates trial was identical to the isolated ingestion of CAF and nitrates. In the case of nitrates, the use of any dietary supplement was considered, mainly concentrated beetroot juice (CBJ). The outcomes examined were those related to exercise performance and physiological responses, such as heart rate, VO_2_ muscle efficiency and lactate thresholds. Finally, the design of the included studies had to be at least a single-blind randomized controlled trial. The following studies were excluded: animal or in vitro studies, studies conducted in diseased or injured subjects or in sedentary people, exclusive supplementation with CAF or nitrates alone or no comparison with these components separately, use of other performance-enhancing techniques or supplements, studies in which the dose or time of supplementation was not specified, articles for which full text was not available, unblinded studies, reviews, opinion articles, editorials and case reports.

### 2.2. Literature Search

Records were retrieved by searching for studies using the databases PubMed, Web of Science, Medline Complete, CINAHL and SPORTDiscus. The terms used for the literature search were: concept 1 (nitrate OR beetroot) AND concept 2 (caffeine) AND concept 3 (ergogenic* OR exercise* OR sport*). All articles published until 3 June 2024 were considered. The search was conducted without any publication year restriction and no filters were used. All titles and abstracts from the search were downloaded to a Microsoft Excel spreadsheet and manual cross-referencing was performed to identify duplicates.

### 2.3. Study Selection

A two-stage search strategy was carried out after duplicates were removed. Firstly, based on reading titles and abstracts, articles that did not meet the eligibility criteria were excluded. Secondly, after reviewing the full texts of the remaining articles, those that did not meet the inclusion criteria were removed. Rayyan^®^ software was used for study selection.

### 2.4. Data Extraction

The subsequent information was extracted from the selected studies: study source (authors and year of publication), experimental design (type of study), participants’ characteristics (sample size, different supplementation groups, gender, age, sports discipline), supplementation characteristics (type, dose and timing) and type of exercise (test performed for evaluation, discipline, time or distance, bouts, rest type and time). Given that caffeine and nitrates have shown independent effects on different types of aerobic and anaerobic metabolism, and therefore on different types of physical performance, a wide range of variables have been collected to cover the potential and unexplored effects of concomitant intake on physiological variables (HR, VO_2_, VCO_2_, RER, cortisol, HbO_2_, TSI) and performance (TT, TTE, RPE, power, CMJ, CMJAS, YYR1).

### 2.5. Quality and Risk of Bias Assessment

The quality of each investigation was assessed following Cochrane Collaboration Guidelines. The Cochrane Risk of Bias tool for randomized clinical trials assesses seven domains: sequence generation and allocation concealment (selection bias), blinding of participants and personnel (performance bias), blinding of outcome assessment (detection bias), incomplete outcome data (attrition bias), selective reporting (reporting bias) and other sources of bias (other bias). Risk of bias was categorized as low, high or unclear. The application of the risk of bias tool for the included studies was performed by two separate authors (LG and HPG) and disagreements were resolved through discussion.

### 2.6. Statistical Analysis

Meta-analyses comparing the effect of ingestion of CAF+nitrates vs. CAF alone and nitrates alone were carried out using standardized mean differences (SMDs) estimated by Hedges’ *g* and their respective 95% confidence intervals (95% CIs). For each outcome, the SMD was calculated using mean and standard deviation values from CAF+nitrates vs. CAF alone and nitrates alone, the sample size from each study and the correlations between the trials. Given that none of the studies reported correlation values, a 0.5 correlation was assumed for all trials per recommendations by [9]. For each variable, a pairwise comparison of CAF+nitrates with either CAF alone or nitrates alone was performed for a total of three meta-anlyses for each variable. The magnitude of the effect of CAF+nitrates vs. either CAF alone or nitrates alone in each outcome was interpreted by using the following SMD scale: <0.2 (trivial); 0.2–0.6 (small); 0.6–1.2 (moderate); 1.2–2.0 (large); 2.0–4.0 (very large); and >4.0 (extremely large). For each outcome, a minimum of two studies were required to perform the meta-analysis [10]. Heterogeneity was assessed using the I^2^ statistic and interpreted as: 0–40% (might not be important); 30–60% (may represent moderate heterogeneity); 50–90% (may represent substantial heterogeneity); and 75–100% (considerable heterogeneity). All meta-analyses were performed using the random effects model. The statistical significance threshold was set at *p* <  0.05 for all statistical analyses. The data analyses were performed using Review Manager (Version 5.4, Copenhagen, Denmark) [11].

## 3. Results

### 3.1. Study Selection

**Figure 1:**
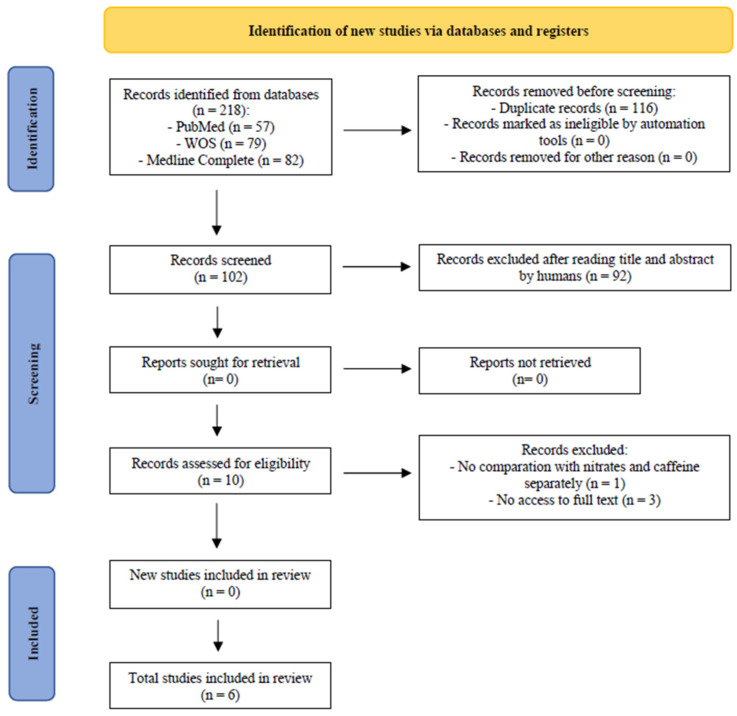
Flow diagram of literature search according to PRISMA guidelines.

Through the databases, 218 articles were identified. Before screening, 116 duplicate records were removed, leaving 102 records, which were screening by title and abstract, and 92 of them were eliminated. There were no articles sought for retrieval, so 10 reports were assessed for eligibility. Of these, four studies were excluded, leaving six randomized placebo-controlled trials for this review (Figure 1).

### 3.2. Characteristics of the Studies

The six randomized controlled trials included had a crossover and double-blind experimental designs. The total number of participants was 95 (67 men and 28 women). Only three of the included articles included women as part of the study sample [12,13,14]. In all articles the sample size was less than 25 participants. The sports disciplines represented were cycling [12,14,15], triathlon [12,14], running [13] and football (soccer) [16]. Castillo et al. (2021) [17] included a sample categorized as an active population without specifying any sport discipline. The age of the participants was 20 to 40 years of age.

In all included studies, CAF supplementation was acute, of which only Handzlik et al. (2013) [15] administered a dose of less than 3 mg/kg body mass. The dose used by the remaining five was 3–6 mg/kg body mass [12,13,14,16,17]. All six studies administered CAF between 10 and 75 min prior to the exercise test. Only Lane et al. (2014) [14] divided CAF supplementation into two doses. Two studies supplemented with CAF as a beverage [15,17], three studies used CAF capsules [12,13,16] and one of them used a caffeinated chewing gum [14]. Nitrate supplementation was also administered acutely for all studies with doses between 6.4 and 8.4 mmol NO_3_^−^. Two of the studies divided the nitrates dose into two intakes, 75 and 150 min before the test [14,15] and 8–12 h and 2 h before [14]. The remaining four administered nitrates supplementation in a single intake 2,5 h before the test [12,13,16,17]. In all studies beet juice concentrate was consumed, except for that by Berjisian et al. (2022) [16] who used a nitrate-containing beverage. The characteristics of the studies reviewed are summarized in Table 1.

### 3.3. Quality and Risk of Bias Assessment

**Figure 2:**
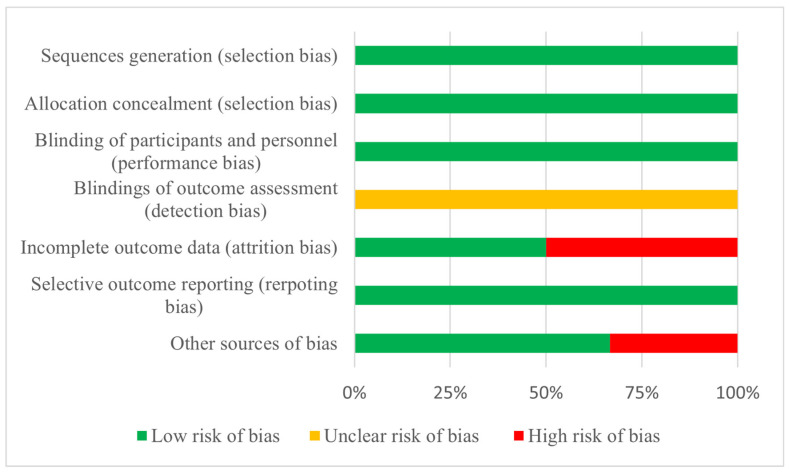
Risk of bias.

In all the studies included in this review, randomized sequence generation and allocation concealment were classified as low risk of bias, as was the case for blinding of participants and personnel. Meanwhile, for blinding of outcome assessment the risk of bias was categorized as unclear. For attrition bias, two articles were categorized as high risk [13,16] and the remaining three as low risk [12,15,17]. All studies were categorized as low risk of reporting bias. Finally, for other sources of bias, two articles were described as high risk [13,14] and the remaining four as low risk of bias [12,15,16,17]. This information is detailed in Table 2 and Figure 2.

### 3.4. Results of Individual Studies

Table 3 presents a summary of the finding of each of the studies included in the systematic review.

#### 3.4.1. Time Trial Performance

Four studies assessed time trial (TT) performance, three of them using a TT of a specific distance [12,13,14] and Handzlik and Gleeson [15] using a time to exhaustion test (TTE) at 80% of VO_2max_. Two studies found no significant differences, while the remaining two found that groups consuming caffeine completed the test in a shorter time than those consuming isolated nitrates or PLA (*p* < 0.05).

On the other hand, Berjisian et al. [16] evaluated the distance covered in a Yo-Yo Intermittent Recovery Test 1 (YYIR1). No significant differences were found between the groups.

#### 3.4.2. Heart Rate

Five of the included studies assessed HR [12,13,14,15,16]. Of these, only the study conducted by Glaister et al. [12] identified differences between groups, with a higher HR observed in CAF consumption group compared to those ingesting nitrates and PLA (*p* < 0.05).

#### 3.4.3. Oxygen Uptake

The assessment of VO_2_ was conducted in three studies [12,13,15], but none of them identified any significant differences between the supplementation groups. Furthermore, the same researchers evaluated RER to estimate fat and carbohydrate oxidation. Only Glaister et al. [12] observed elevated RER values in groups that consumed CAF versus nitrates and PLA (*p* < 0.01).

#### 3.4.4. Power Output

In a cycling TT, Lane et al. [14] examined mean power and observed higher results for the CAF and CAF+nitrate groups than for the rest (*p* < 0.01). Besides that, Glaister et al. [12] investigated power output in a TT and also found significant results for the CAF groups (*p* < 0.05), but no effects on cadence were perceived.

#### 3.4.5. Perceived Effort

Perceived exertion was assessed using RPE [13,14,15]. Three studies analyzed this variable, however, only Handzlik and Gleeson [15] found a decrease in RPE for the CAF+nitrates group compared to PLA.

#### 3.4.6. Countermovement Jump

CMJ was evaluated by Castillo et al. and Berjisian et al. [16,17] with no significant differences for either of them. 

### 3.5. Meta-Analyses

**Figure 3:**
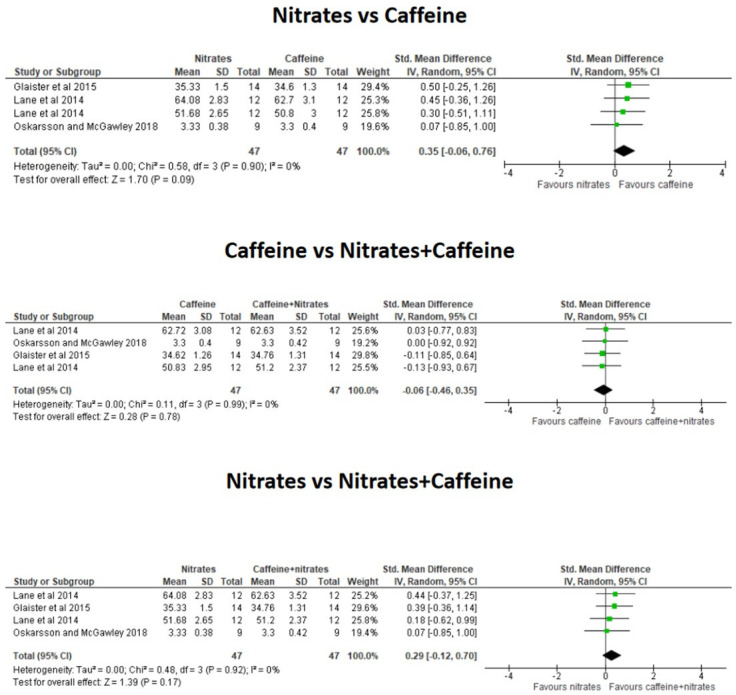
Effect of the co-ingestion of caffeine and nitrates compared to the isolated ingestion of caffeine and nitrates on exercise time trials. The green squares represent standardized mean differences with 95% confidence intervals (CI) for each study included in the meta-analysis. The diamond at the bottom presents the pooled effect obtained from all studies included in the meta-analysis [12,13,14].

**Figure 4:**
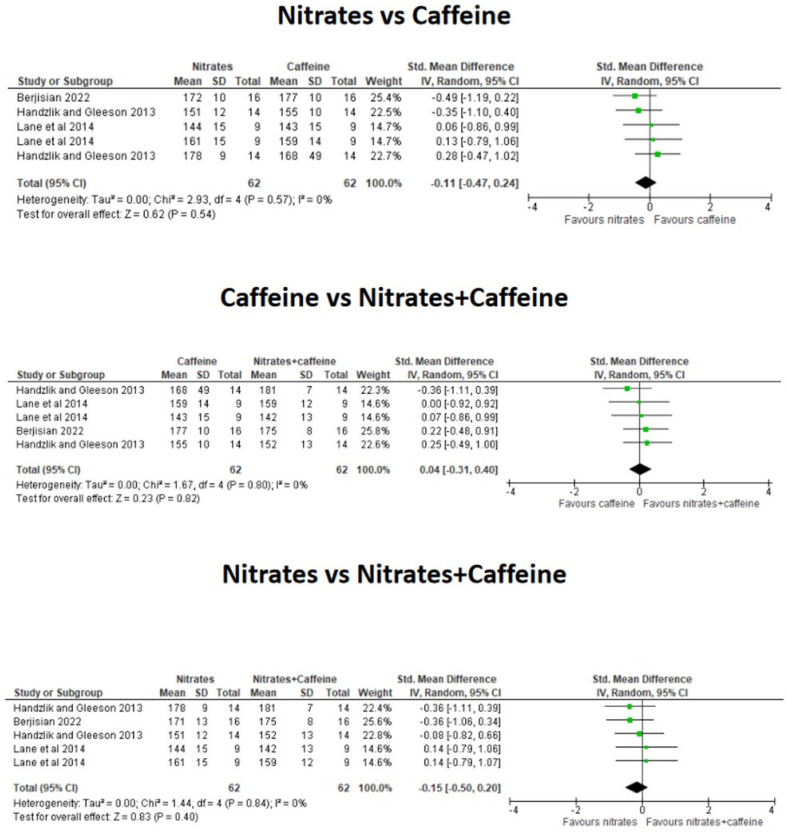
Effect of the co-ingestion of caffeine and nitrates compared to the isolated ingestion of caffeine and nitrates on heart rate during submaximal exercise. The green squares represent standardized mean differences with 95% confidence intervals (CI) for each study included in the meta-analysis. The diamond at the bottom presents the pooled effect obtained from all studies included in the meta-analysis [14,15,16].

**Figure 5:**
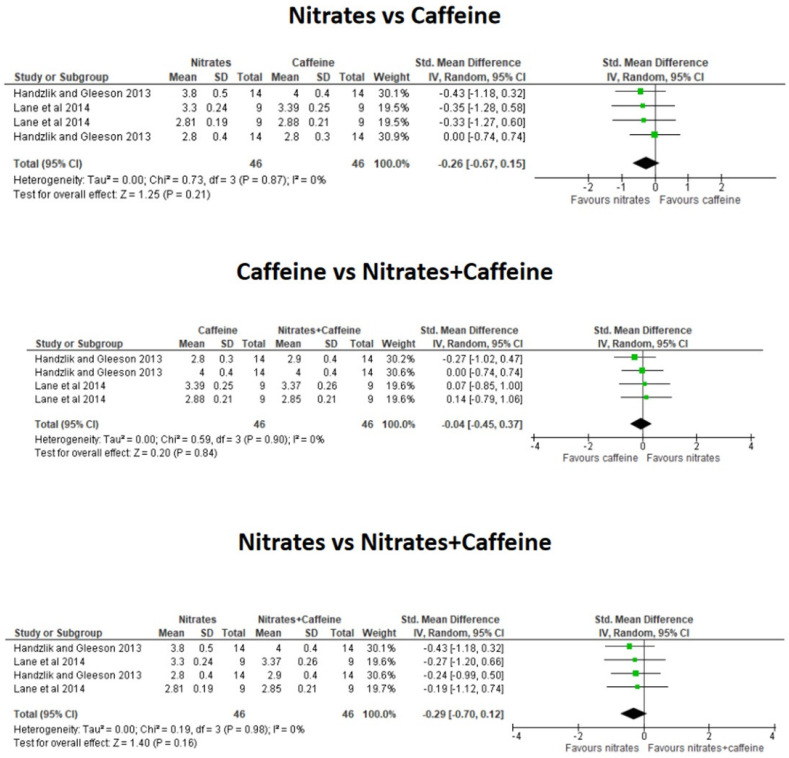
Effect of the co-ingestion of caffeine and nitrates compared to the isolated ingestion of caffeine and nitrates on oxygen uptake during submaximal exercise. The green squares represent standardized mean differences with 95% confidence intervals (CI) for each study included in the meta-analysis. The diamond at the bottom presents the pooled effect obtained from all studies included in the meta-analysis [14,15].

The meta-analysis revealed that the combination of CAF+nitrates did not significantly reduce the time to complete the exercise trials over the ingestion of CAF alone (*g* = −0.06; 95% CI = −0.46 to 0.35; *p* = 0.78) or the ingestion of nitrates supplementation alone (*g* = 0.29; 95% CI = −0.12 to 0.70; *p* = 0.17, Figure 3). There was a tendency for a better TT performance with isolated CAF than isolated nitrates alone (*g* = 0.35; 95% CI = −0.06 to 0.76; *p* = 0.09) although it did not reach the level of statistical significance. The combination of CAF+ nitrates did not affect HR during steady-state exercise trials over the ingestion of CAF alone (*g* = 0.04; 95% CI = −0.31 to 0.40; *p* = 0.80) or nitrates alone (*g* = −0.15; 95% CI = −0.50 to 0.20; *p* = 0.40, Figure 4). Additionally, the effect of either CAF or nitrates alone produced a comparable effect on HR during exercise (*g* = −0.11; 95% CI = −0.47 to 0.24; *p* = 0.54). Likewise, the combination of CAF+ nitrates did not affect VO_2_ during steady-state exercise over the ingestion of CAF alone (*g* = −0.04; 95% CI = −0.45 to 0.37; *p* = 0.84) or nitrates alone (*g* = −0.29; 95% CI = −0.70 to 0.12; *p* = 0.16). The effect of either CAF or nitrates alone produced a comparable effect on oxygen uptake during exercise (*g* = −0.26; 95% CI = −0.67 to 0.15; *p* = 0.21, Figure 5).

## 4. Discussion

The aim of this systematic review was to analyze the effects of co-supplementation of CAF and nitrates on sports performance in comparison to the isolated ingestion of these substances. Overall, the meta-analysis of the studies included in this review revealed that the co-ingestion of CAF+nitrates did not provide additional benefits on exercise performance during TT and did not affect heart rate or oxygen uptake during submaximal exercise over the isolated ingestion of these substances. Additionally, only one out of the six studies included showed that the combination of these compounds had a greater effect than the ingestion of them individually [15]. Collectively, the information provided by these investigations suggests that the co-ingestion of CAF+nitrates did not offer further benefits from the isolated intake of CAF and nitrates. From a practical perspective for sports scientists and sports nutritionists, the use of either CAF or nitrates should be recommended depending on the type of effect desired, central (CAF) or peripheral (nitrates), and on the characteristics of the sports while the combination of these substances is not generally recommended due to the lack of additional/synergistic effect.

In the review carried out by Dominguez et al. (2017) [18] about the effect of nitrates on exercise performance, a section was used to discuss the effect of combining nitrates and caffeine on performance and cardiorespiratory endurance. In the aforementioned review, the authors discussed three studies that were included and concluded in their analysis of them that CAF+nitrates combination did not provide further benefits with respect to isolated ingestion of these substances. As in the current review, the results of the individual studies generally point toward a benefit of CAF+nitrates on parameters such as average power, TT performance and time to exhaustion, although the effect of this combination is not greater than the ergogenic effect of these same substances separately. The current investigation is novel because, in addition to the summary of studies, we have meta-analyzed the effect of the substances. The low number of studies devoted to studying the synergistic effect of CAF+nitrates and the different approaches used precluded meta-analyzing a wide variety of performance protocols or physiological variables, but with the current evidence we were able to ascertain that the co-ingestion of CAF+nitrates did not reduce time to complete a distance and did not affect heart rate or oxygen uptake during steady-state exercise. Therefore, both the systematic review and the meta-analysis of studies coincide to indicate that combining CAF and nitrates does not provide synergistic effects.

Even though CAF is a supplement whose effect on improving performance in strength and endurance exercises has been widely demonstrated for several decades by multiple systematic reviews and meta-analyses [19,20] two of the studies included in this systematic review observed no effect when administering this supplementation [12,14] in the tests performed (YYIR1 test and treadmill test consisting of 5 min at 70% VO_2max_, 5 min at 80% VO_2max_ and 1 km TT at own pace). Given that CAF has been shown to improve performance in both high-intensity [21,22] and endurance [23,24,25] tests, it is possible that the lack of erogenicity is not related to the supplement itself and may be due to external factors such as nutritional status, sleep pattern, activity level, training status and sample size [26]. Another possible explanation is that certain subjects are accustomed to the consumption of CAF and they may develop tolerance to the ergogenic effect of acute caffeine intake as new adenosine receptors are created with chronic intake of caffeine [27]). Briefly, crossover studies including participants that have undergone a controlled habituation to caffeine through daily intake of caffeine showed that caffeine is more ergogenic the first day of intake and then there is a progressive ergogenic response to acute caffeine intake [6,28,29]. So, it is possible that the lack of effect to caffeine is associated with tolerance developed by chronic intake, although this is only a hypothesis as information about habituation to caffeine was rarely included in the studies analyzed. Anyway, the systematic reviews and meta-analysis that have summarized the outcomes of the research investigating the effect of acute caffeine intake on sport-specific physical tests have confirmed, almost unanimously, that caffeine increases several aspects of exercise performance such as aerobic and anaerobic activities, coinciding with the outcomes of most of the investigations included in this systematic review.

The effect of nitrates was only demonstrated by two of the studies included in the review [15,17]. There are many factors that influence the ergogenic component of nitrate supplementation, especially training status and intensity of exercise test. Highly trained subjects usually require higher doses of nitrates to achieve the same effect, for two reasons [30]. First is that they have higher NOS enzyme activity, making the NO_3_^−^–NO_2_^−^–NO pathway less important in NO production [31]. Second, these subjects have higher plasma NO_2_^−^ concentrations than sedentary individuals [32], so response to a standard dose of nitrate may be diminished. Instead, NO_2_^−^ is reduced to NO under acidic and hypoxic conditions, and trained subjects have better muscle capillarity, which could minimize muscle tissue hypoperfusion during exercise and reduce NO production [30]. There will not be much NO production at low exercise intensities, in which the muscle is well oxygenated, and pH does not drop significantly, so nitrate supplementation will only be effective at high intensities [30]. For example, in a study by [33], subjects performed 4 km and 16 km TTs at intensities of approximately 98% and 95% VO_2max_ and they detected an improvement in test performance with nitrate supplementation. However, in most of the studies included in this review, which did not observe effects of this supplementation, exercise intensities were less than 90% VO_2max_ [12,13,14]. In addition, in high-intensity exercise there is a greater recruitment of type II fibers. Evidence suggests that nitrate effects blood flow [34], muscle strength, calcium handling and contractile function of type II fibers [35]. Therefore, endurance athletes, who have a lower proportion of these fibers in their musculature, may have a diminished response to this ergogenic aid [36]. Previous studies suggest that there may be “responders” and “non-responders” to nitrate supplementation [37,38]. For example, in Lane et al.’s (2014) study [14], 2 of the 26 participants had better performance with CBJ consumption, both in isolation and together with CAF. Likewise, in the study by Oskarsson and McGawley (2018) [13] there is a similar pattern, as three of the nine participants showed an improvement in running economy and two of them an enhancement in test performance when supplemented with CBJ. A possible line of future research could be the study of factors that determine these interindividual differences and whether there is a genetic component that regulates whether a subject is a responder to nitrate supplementation.

It is noteworthy that five of the six included studies examined HR [12,13,14,15,16]. However, the results of the meta-analysis indicated that there were no statistically significant differences in HR associated with any type of supplementation. This may be interpreted in a number of ways. Firstly, it is established that CAF acts on the autonomic nervous system by increasing catecholamine secretion, which in turn produces an increase in HR [19]. However, it is possible that the vasodilator effects of nitrates may counteract this effect, which is why no differences are found with CAF+nitrates supplementation. Conversely, although it is possible that caffeine exerts a direct effect on heart rate, research on the effects of caffeine in fixed-intensity submaximal exercise generally shows no effect [39,40,41]. This would explain the ineffectiveness of supplementation on HR.

In another vein, some of the studies included in this review [12,13,15] analyze variables such as gas exchange variables (VO_2_ and VCO_2_), fat and carbohydrate oxidation or RER, which not only have to do with sports performance but also with metabolic flexibility. There were no significant differences between groups for all these parameters in the three studies with exception of Glaister et al. (2015) [12], who found higher RER values for the CAF group in comparation with placebo and nitrates groups.

The concept of metabolic flexibility was established by Kelley and Mandarino (2000) [42] and it is defined as “the ability of an organism to respond or adapt according to changes in metabolic or energy demand as well as prevailing conditions or activity” [43]. At the molecular level, metabolic flexibility is understood as the configuration of metabolic pathways that manage the sensing, absorption, transport, storage and utilization of nutrients [43]. In healthy individuals, metabolic flexibility allows for changes in glucose and fatty acid oxidation during acute exercise depending on exercise intensity and duration. The contribution of fatty acid oxidation to total energy intake increases with exercise duration. On the other hand, in high-intensity exercise, muscle fibers depend on oxidative phosphorylation to obtain ATP [44] and as the intensity becomes higher, anaerobic glycolysis is prioritized [43]. In relation to this, the following question arises. An improvement in metabolic flexibility could allow a greater oxidation of fatty acids at higher intensity, which would make it possible to maintain this intensity for a longer time, given that fatty acid reserves are greater than glycogen reserves, with the improvement in performance that this would entail. The effect of CAF and nitrates on the improvement of metabolic flexibility, and the consequent improvement in performance, may be a very interesting line of study for future research.

Finally, this review is not without limitations. The first is that the literature found so far on CAF and nitrate supplementation is scarce, only six studies related to the subject were found. Secondly, it is important to mention that the participation of women in these studies is much lower than that of men. Of the 95 subjects who participated in total, only 28 were women, and a single study [14] included the same number of men as women among its participants. This makes it difficult to draw conclusions for both sexes.

Special treatment needs to be given to the research of Lane et al. (2014) [14], given that participants were provided with carbohydrates before and during the test, in the form of gels and sports drinks. This manner of supplementation is widely studied and is associated with improved performance, especially in endurance training [45,46]. However, in this study, carbohydrate supplementation was administered to replicate optimal competition conditions. Furthermore, since carbohydrates were administered to all supplementation groups, we decided to include this research in our review, as we considered that carbohydrate consumption before and during the test would not bias the results obtained in the test.

Although all studies included in the review were double-blinded, randomized controlled studies, the doses of caffeine and nitrates and the forms of administration (mostly capsules for CAF and mostly CBJ for nitrates) were diverse among studies, which makes it difficult to determine if the dose or and via of administration affected the lack of synergistic effect when combining both substances. Last, from a methodological perspective, all the studies were classified as low risk of bias. Therefore, the outcomes of the current review are supported by high-quality studies, which strengthens the main conclusions [14,45,46]. However, due to the small samples of studies related to this topic, these conclusions cannot be taken as determinative.

## 5. Conclusions

In conclusion, the co-ingestion of CAF+nitrates does not appear to provide additive or synergistic benefits on exercise performance compared the isolated ingestion of either CAF or nitrates. From a practical perspective, no additional advantages were observed with the co-ingestion of these substances, and their combination is not generally recommended. However, considering the scarcity of studies and the variability in caffeine doses, ranging from below the expected effective threshold (less than 3 mg/kg) to 6 mg/kg, where side effects may occur, it is suggested to await more relevant studies that will enable conclusions with greater practical implications.

## Figures and Tables

**Table 1:** Characteristics of the studies reviewed.

Study	Design	Blinding	Participants	Sport Discipline	Parcipants Age (M ± SD)	CAF	Nitrates
Handzlik and Gleeson (2013) [15]	Crossover	Double blind	14 men	Cycling	22 ± 3 years	Beverage (1 period)	CBJ (2 periods)
Lane et al. (2014) [14]	Crossover	Double blind	12 men, 12 women	Cycling and triathlon	31 ± 7 years (male) 28 ± 6 years (female)	Gum (2 periods)	CBJ (2 periods)
Glaister et al. (2015) [12]	Crossover	Double blind	14 women	Cycling and triathlon	31 ± 7 years	Capsule (1 period)	CBJ (1 period)
Oskarsson et al. (2018) [13]	Crossover	Double blind	7 men, 2 women	Running	30.4 ± 6.3 years (male) 31.5 ± 9.2 years (female)	Capsule (1 period)	CBJ (1 period)
Castillo et al. (2021) [17]	Crossover	Double blind	16 men	NS	22.8 ± 4.9 years	Beverage (1 period)	CBJ (1 period)
Berjisian et al. (2022) [16]	Crossover	Double blind	18 men	Soccer	19.8 ± 2.2 years	Capsule (1 period)	Nitrate beverage (1 period)

Abbreviations: not specified (NS); concentrated beetroot juice (CBJ), mean (M), standard derivation (SD).

**Table 2:** Risk of bias of the studies included.

Study	Sequence Generation (Selection Bias)	Allocation Concealment (Selection Bias)	Blinding of Participants and Personnel (Performance Bias)	Blindings of Outcome Assessment (Detection Bias)	Incomplete Outcome Data (Attrition Bias)	Selective Outcome Reporting (Reporting Bias)	Other Sources of Bias
Handzlik and Gleeson (2013) [15]	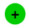	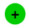	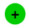	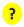	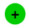	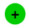	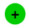
Lane et al. (2014) [14]	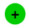	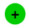	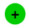	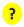	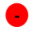	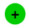	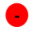
Glaister et al. (2015) [12]	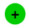	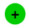	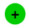	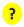	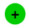	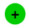	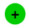
Oskarsson et al. (2018) [13]	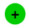	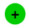	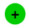	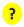	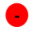	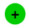	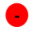
Castillo et al. (2021) [17]	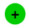	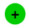	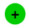	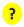	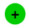	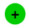	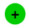
Berjisian et al. (2022) [16]	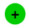	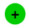	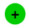	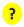	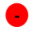	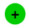	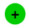

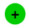
 Low risk of bias 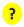 Unclear risk of bias 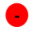 High risk of bias.

**Table 3:** Results and characteristics of individual studies.

	Experimental Design	Subjects	Supplementation	Performance Trials	Variables Measured	Performance Results	Physiological Results
Handzlik and Gleeson (2013) [15]	Double-blind RCT with a crossover design (CAF, CBJ, CAF + CBJ, PLA)	14 male cyclists (22 ± 3 years old)	CAF 0.5 g/kg (75 min before) CBJ 140 mL (8 mmol NO_3_^−^) (70 mL 150 min before and 70 mL 75 min before) PLA	30 min at 60% VO_2max_ in cycle ergometer TTE trial at 80% VO_2max_	Performance variables TTE RPE (each 5 min at 80% VO_2max_) Physiological variables Mean HR VO_2_ and VCO_2_ Carbohydrate and fat oxidation RER Salivary NO_3_^−^ and NO_2_^−^ Salivary cortisol	TTE: CAF = CBJ = CAF + CBJ = PLA RPE: CBJ + CAF < CAF = PLA = CBJ (in15 min TTE trial)	Mean HR, VO_2_ and VCO_2_, carbohydrate and fat oxidation and RER CAF = CBJ = CAF + CBJ = PLA Salivary NO_3_^−^ and NO_2_^−^ Pre-SUP < post-SUP: CAF and CAF + CBJ Salivary cortisol: Post-exercise > during exercise > pre-exercise
Lane et al. (2014) [14]	Double-blind RCT with a crossover design (CAF, CBJ, CAF + CBJ, PLA)	14 male (31 ± 7 years old) and 12 female (28 ± 6 years old) cyclists or triathletes	CAF 3 mg/kg (2 mg/kg 40 min before and 1 mg/kg 10 min before) CBJ 280 mL (16.8 mmol NO_3_^−^) (140 mL 8–12 h before and 140 mL 120 min before) PLA	43.83 km (male) or 29.35 km (female) TT on cycle ergometer	Performance variables Mean power TT completion time Physiological variables BM Plasma caffeine Plasma NO_3_^−^ and NO_2_^−^ HR RPE	Mean power CAF + CBJ and CAF > PLA = CBJ TT completion time CAF + CBJ and CAF < PLA = CBJ RPE CAF = CBJ = CAF + CBJ = PLA	BM Pre-TT = post-TT Plasma caffeine Pre-SUP < post-SUP: CAF and CAF + CBJ Plasma NO_3_^−^ and NO_2_^−^ Pre-SUP < post-SUP: CAF and CAF + CBJ HR CAF = CBJ = CAF + CBJ = PLA
Glaister et al. (2015) [12]	Double-blind counterbalanced RCT with a crossover design (CAF, CBJ, CAF + CBJ, PLA)	14 female cyclists or triathletes (31 ± 7 years)	CAF 5 mg/kg 1 h before CBJ 70 mL (7.3 mmol NO_3_^−^) 2.5 h before PLA	20 km TT on a racing bicycle seated on a motor-braked turbo trainer	Performance variables Power output Cadence RPE Physiological variables Plasma caffeine Plasma NO_3_^−^ and NO_2_^−^ HR VO_2_ RER TSI, [HbO_2_], [HHb], iEMG	Power output CAF = CAF + CBJ > CBJ = PLA Cadence and RPE CAF = CBJ = CAF + CBJ = PLA	Plasma caffeine Pre-SUP < post-SUP: CAF and CAF + CBJ Plasma NO_3_^−^: Pre-SUP < post-SUP: CAF and CAF + CBJ Plasma NO_2_^−^: Pre-SUP < post-SUP: CBJ CAF = CAF + CBJ > CBJ = PLA RER CAF > PLA = CBJ VO_2_, tissue oxygenation, iEMG CAF = CBJ = CAF + CBJ = PLA
Oskarsson et al. (2018) [13]	Double-blind counterbalanced RCT with a crossover design (CAF, CBJ, CAF + CBJ, PLA)	7 male (30.4 ± 6.3 years) and 2 female (31.5 ± 9.2 years) endurance runners	CAF 4–6 mg/kg 45 min before CBJ 70 mL (7.3 mmol NO_3_^−^) 2.5 h before PLA	Treadmill running tests: 5 min at 70% VO_2max_ and 5 min at 80% VO_2max_ 1 km self-paced TT	Performance variables Running economy RPE at submaximal and maximal test 1 km running TT Physiological variables VO_2_ RER HR Max. HR Peak blood lactate	All variables CAF = CBJ = CAF + CBJ = PLA	All variables CAF = CBJ = CAF + CBJ = PLA
Castillo et al. (2021) [17]	Double-blind RCT with a crossover design (CAF, CBJ, CAF + CBJ, PLA)	16 male endurance athletes (22.8 ± 4.9 years)	CAF 6 mg/kg 35 min before CBJ 140 mL 2 h and 30 min before PLA	Half-squat power test: 4 × 8 all-out repetitions with 3 min rest between them (using differences inertial loads) CMJ Before, 30 s after and 180 s after	Performance variables Total mean power in half squat power test CMJ height	Total mean power CAF = CAF + CBJ > PLA No differences between groups in CMJ height	
Berjisian et al. (2022) [16]	Double-blind RCT with a crossover design (CAF, CBJ, CAF + CBJ, PLA)	16 semi-professional male soccer players (19.8 ± 2.2 years)	CAF 5 mg/kg 60 min before CBJ 60 mL (6.4 mmol NO_3_^−^) 2.5 h before * PLA	YYIR1 2 × 20 m shuttle run at a gradually progressive speed with 10 s active recovery CMJAS 3 jumps with 30 s rest between them Immediately before and after YYIR1 Stroop word-color test	Performance variables Distance covered during YYIR1 CMJAS jump height CMJAS power output Stroop test performance RPE Physiological variables HR GI symptoms	Distance covered during YYIR1, maximum CMJAS height or power output, Stroop test performance and RPE CAF = CBJ = CAF + CBJ = PLA	HR max CAF = CBJ = CAF + CBJ = PLA GI symptoms Upper GI symptoms: CAF = CBJ = CAF + CBJ = PLA Sum scores: CAF + CBJ > PLA

Abbreviations: body mass (BM), caffeine (CAF), concentrated beetroot juice (CBJ), countermovement jump (CMJ), countermovement jump with arm swing (CMJAS), deoxyhemoglobin concentration change ([HHb]), gastrointestinal (GI), heart rate (HR), integrated electromyography (iEMG), nitrates (NO_3_^−^), nitrites (NO_2_^−^), maximal oxygen uptake (VO_2max_), mean carbon dioxide production (VCO_2_), mean oxygen uptake (VO_2_), oxyhemoglobin concentration change ([HbO_2_]), placebo (PLA), randomized controlled trial (RCT), rate of perceived exertion (RPE), respiratory exchange ratio (RER), supplementation (SUP), time to exhaustion (TTE), time trial (TT), tissue saturation index (TSI), Yo-Yo Intermittent Recovery Test level 1 (YYIR1).* In a bottle which contained 500 mg L-arginine and L-ornithine too.
